# Systemic Bone Loss and Periodontal Disease: An Updated Review of a Bidirectional Association

**DOI:** 10.3390/dj14010070

**Published:** 2026-01-22

**Authors:** Abdulkareem A. Alhumaidan, Ahmed Elakel

**Affiliations:** Department of Preventive Dental Sciences, College of Dentistry, Imam Abdulrahman Bin Faisal University, P.O. Box 1982, Dammam 31441, Saudi Arabia

**Keywords:** systemic bone loss, osteoporosis, periodontal disease, periodontitis, alveolar bone loss, bone mineral density, bidirectional relationship

## Abstract

**Background:** Systemic bone loss, particularly osteoporosis, and periodontal disease are highly prevalent chronic conditions that share common risk factors and biological pathways. Increasing evidence suggests a bidirectional relationship between these conditions; however, findings remain heterogeneous and evolving. **Objective:** This review aims to evaluate and update current evidence on the bidirectional association between systemic bone loss and periodontal disease, with emphasis on underlying mechanisms and clinical implications. **Methods:** A narrative review of the literature was conducted using major electronic databases, focusing on human studies evaluating the relationship between osteoporosis or systemic bone loss and periodontal disease. Relevant experimental, clinical, and epidemiological studies were included. **Results:** Most studies support an association between reduced bone mineral density and increased severity of periodontal disease, including greater alveolar bone loss and attachment loss. Conversely, periodontal inflammation may contribute to systemic bone remodeling through inflammatory mediators. However, variability in study design, diagnostic criteria, and confounding factors limits definitive conclusions. **Conclusions:** Current evidence supports a bidirectional association between systemic bone loss and periodontal disease. Greater interdisciplinary awareness is warranted, and future well-designed longitudinal studies are needed to clarify causality and inform preventive and therapeutic strategies.

## 1. Introduction

Osteoporosis is a systemic skeletal disorder characterized by reduced bone density and microarchitectural deterioration of bone tissue, defined by a bone mineral density (BMD) T-score of 2.5 standard deviations or more below the mean BMD of a healthy young adult. Osteopenia represents an intermediate condition, with BMD values between 1 and 2.5 standard deviations below normal [1]. The degree of BMD reduction varies according to age and sex, with a higher prevalence observed among older individuals, particularly postmenopausal women. Several lifestyle and systemic factors contribute to reduced BMD and osteoporosis, including alcohol consumption, tobacco use, diabetes, vitamin D deficiency, low body mass, and physical inactivity [2,3].

Periodontitis and osteoporosis are both highly prevalent chronic inflammatory conditions that pose significant public health challenges, especially in aging populations. Periodontal diseases comprise a spectrum of inflammatory conditions affecting the dentogingival tissues, characterized by dysbiosis and a host-mediated inflammatory response that leads to destruction of the supporting structures of the teeth [4]. Given the shared inflammatory nature and overlapping risk factors, increasing interest has been directed toward understanding the potential interaction between periodontal diseases and bone mineral disorders. However, evidence supporting a direct association between the two conditions remained conflicting until the early 2000s [5,6].

Systemic conditions such as osteoporosis may influence the progression of periodontitis by modifying the host response and bone metabolism, rather than acting as direct causative factors. Although osteoporosis has been associated with deterioration of the periodontal supporting tissues, it does not produce distinctive periodontal clinical signs. Consequently, it is more appropriately considered a modifying or risk factor rather than a primary etiologic agent. Notably, the 2017 World Workshop task group concluded that osteoporosis is significantly associated with increased prevalence and severity of radiographic alveolar bone loss, while no consistent association was established with other clinical parameters of periodontitis [7,8,9,10].

## 2. Methods

This article is a narrative review aimed at providing an updated synthesis of the evidence regarding the bidirectional relationship between systemic bone loss, particularly osteoporosis, and periodontal disease. A literature search was conducted using major electronic databases, including PubMed/MEDLINE (National Library of Medicine, Bethesda, MD, USA), Scopus (Elsevier, Amsterdam, The Netherlands), and Web of Science Core Collection (Clarivate, Philadelphia, PA, USA).

The search included articles published up to December 2024 and was limited to studies published in English. Keywords and search terms included combinations of osteoporosis, systemic bone loss, bone mineral density, periodontal disease, periodontitis, alveolar bone loss, and jaw bone. In addition, reference lists of relevant articles were manually screened to identify further pertinent studies.

The review primarily included human clinical, epidemiological, and observational studies, as well as selected experimental and translational studies that contributed to understanding the biological mechanisms linking osteoporosis and periodontal disease. Relevant systematic reviews and meta-analyses were used to contextualize findings where appropriate. Case reports, conference abstracts, and non–peer-reviewed sources were excluded.

Given the narrative nature of this review, no formal quality assessment or quantitative synthesis was performed. Instead, the evidence was critically summarized and interpretively synthesized to highlight consistent findings, biological plausibility, clinical relevance, and gaps in the existing literature.

## 3. Key Findings on the Osteoporosis–Periodontitis Association

### 3.1. Diagnostic Evidence

Several diagnostic techniques are used to assess osteoporosis, with dual-energy X-ray absorptiometry (DXA) considered the gold standard. DXA measures bone mineral density (BMD) at clinically relevant sites, including the lumbar spine and proximal femur. Quantitative computed tomography (QCT) is another modality that allows three-dimensional assessment of bone density and can be used to estimate fracture risk. Osteoporosis is associated with thinning and loss of both trabecular and cortical bone, including the alveolar bone and cortical plates of the jaws.

Numerous studies conducted between 1999 and 2020 have demonstrated an association between systemic osteoporosis and alveolar bone loss. Alveolar bone changes have been evaluated using various radiographic techniques, such as linear measurements of alveolar crest height (ACH) or alveolar bone loss (ABL) on intraoral and panoramic radiographs, mandibular cortical width (MCW) assessment on panoramic radiographs, and digital densitometric analysis of alveolar BMD. MCW, which reflects the thickness of the inferior mandibular cortex, has been shown to correlate strongly with systemic BMD and has been proposed as a potential radiographic risk marker for osteoporosis [10,11,12,13,14,15,16,17,18].

In addition to cortical bone measurements, the mandibular trabecular bone pattern has been reported to be more closely associated with fracture risk than cortical bone density, particularly in females. While trabecular bone connectivity tends to remain relatively stable in males with aging, females often exhibit reduced trabecular connectivity and increased intertrabecular spacing. Due to the difficulty of directly quantifying trabecular architecture, most studies have relied on indirect measures such as alveolar crest height and alveolar BMD, which represent the sites most affected by periodontal bone loss. Consistent correlations have been reported between systemic BMD and both alveolar crest height and alveolar BMD measurements [19,20,21,22].

### 3.2. Mechanism

Osteoporosis and other systemic inflammatory bone diseases are characterized by dysregulated bone remodeling driven by chronic inflammation, increased cytokine release, and activation of the nuclear factor kappa B (NF-κB) signaling pathway. NF-κB activation simultaneously suppresses osteoblast differentiation and promotes osteoclastogenesis, leading to net bone loss. Experimental studies have demonstrated that chemical or genetic suppression of NF-κB signaling can significantly reduce bone loss in conditions such as osteoporosis and arthritis [23,24,25,26].

Several pro-inflammatory cytokines contribute to this process, including tumor necrosis factor-α (TNF-α) and interleukin-1 (IL-1). Therapeutic blockade of TNF-α has been shown to slow systemic and local bone loss, particularly in inflammatory diseases such as rheumatoid arthritis, supporting the role of inflammatory mediators in bone destruction [25,26]. In addition, IL-1 and IL-6 play key roles in both systemic and periodontal bone loss. Elevated levels of IL-1β have been detected in gingival crevicular fluid at sites of periodontal attachment loss in humans. Experimental models further support this association, as animals with pharmacologic or genetic inhibition of IL-1 exhibit reduced bacterial burden and bone loss, whereas transgenic overexpression of IL-1α results in periodontitis with attachment loss and alveolar bone resorption [27].

Beyond cytokine signaling, the complement system also contributes to inflammatory bone loss by amplifying host immune responses. Complement activation enhances NF-κB and activator protein-1 (AP-1) signaling, leading to increased production of IL-6, TNF-α, and IL-1β. Patients with periodontitis and osteoporosis have been reported to exhibit higher levels of activated complement components in gingival crevicular fluid compared with healthy individuals. In contrast, animal models deficient in complement component C3 demonstrate reduced rates of bone loss, further supporting the role of complement activation in bone resorption [28,29,30,31].

In inflammatory bone loss conditions such as osteoporosis and rheumatoid arthritis, osteoblast activity and number are insufficient to counterbalance increased osteoclastic resorption. Inflammatory mediators may impair osteoblast differentiation through multiple mechanisms. For example, TNF-α promotes degradation and suppresses expression of RUNX2, a master transcription factor required for osteoblast differentiation, via upregulation of E3 ubiquitin ligases. Additionally, suppression of components of the NF-κB pathway, such as IKKγ, has been shown to alter AP-1 signaling and negatively affect osteoblastogenesis [23].

## 4. Modifying and Shared Risk Factors

### 4.1. Relation to Aging

Osteoporosis and periodontitis share aging as an important common risk factor. In both males and females, advancing age is associated with increased fracture risk and loss of trabecular bone density, even in the presence of sufficient sex-related hormones. This observation supports the concept that both conditions are influenced by age-related inflammatory processes. Chronic low-grade inflammation, a hallmark of aging, contributes to bone loss through increased oxidative stress, DNA damage, mitochondrial dysfunction, and elevated production of inflammatory cytokines, ultimately leading to increased generation of reactive oxygen species (ROS). Through activation of the NF-κB signaling pathway, oxidative stress suppresses osteogenesis while enhancing osteoclast activity [5,6].

With aging, the bone marrow microenvironment becomes increasingly enriched with pro-inflammatory mediators such as interleukin-1 (IL-1) and interleukin-6 (IL-6). This inflammatory milieu renders bone metabolism more susceptible to osteoimmunological dysregulation, as both immune and bone cells are directly affected by chronic inflammatory signaling. Experimental studies have demonstrated activation of NF-κB signaling in murine models of accelerated aging, further supporting its role in age-related bone loss [32,33].

Epidemiological evidence suggests that aging alone does not fully account for the severity of periodontal destruction. Cross-sectional studies have reported that alveolar bone mass may remain relatively stable after the age of 50, indicating that increased prevalence and severity of periodontitis in older individuals are more likely related to altered host response and increased disease susceptibility rather than aging per se [34].

Supporting this concept, studies in non-human primates have shown that gingival inflammation and periodontal tissue breakdown are associated with significantly higher levels of systemic inflammatory mediators in older animals. Although the precise mechanisms remain incompletely understood, emerging evidence suggests that cellular senescence and cumulative oxidative stress are key age-related processes contributing to both osteoporosis and the progression of periodontitis [35,36].

### 4.2. Relation to Post Menopause

Postmenopausal osteoporosis (PMO) has been implicated in dental osteopenia affecting both jaws, with a more pronounced impact on the mandible. During the postmenopausal period, estrogen deficiency leads to an imbalance in bone remodeling, resulting in progressive reduction in bone mass. These systemic hormonal changes contribute to decreased bone mineral density (BMD), osteopenia, and, in more advanced cases, osteoporosis. Numerous studies have demonstrated a close association between reduced estrogen levels and increased susceptibility to periodontitis, alveolar bone loss, and systemic osteoporosis [37,38,39,40,41,42].

Clinical evidence indicates that postmenopausal women with estrogen deficiency and a history of periodontitis experience more severe periodontal attachment loss. This association is likely attributable to shared etiological and modifying risk factors that influence both osteoporosis and periodontal disease. Systematic reviews and meta-analyses comparing postmenopausal osteoporotic and non-osteoporotic women have consistently reported worse periodontal parameters in osteoporotic postmenopausal groups, including greater attachment loss and alveolar bone resorption [43,44,45].

Estrogen plays a central role in maintaining skeletal homeostasis by regulating the balance between osteoblastic bone formation and osteoclastic bone resorption. In addition to its effects on bone cells, estrogen exerts anti-inflammatory properties by suppressing the activity of key pro-inflammatory cytokines, including interleukin-1 (IL-1), interleukin-6 (IL-6), interleukin-10 (IL-10), and tumor necrosis factor-α (TNF-α). Loss of this regulatory effect following menopause contributes to enhanced inflammatory signaling and increased vulnerability to both systemic and periodontal bone loss [46,47].

### 4.3. Relation to Dietary Intake

Deficiencies in natural resources, particularly calcium and vitamin D, represent important shared risk factors for both periodontitis and osteoporosis. Inadequate vitamin D intake, defined as serum concentrations below 25 nmol/L, reduces intestinal calcium absorption and triggers compensatory skeletal calcium mobilization to maintain calcium homeostasis. Large-scale cross-sectional studies have demonstrated that women with low dietary calcium intake exhibit more severe periodontal disease, whereas the association appears weaker in males [48,49,50,51].

Vitamin D plays a central role in bone metabolism by regulating parathyroid hormone (PTH) secretion, enhancing intestinal calcium absorption, and mediating PTH-induced bone resorption. Beyond its endocrine effects, vitamin D also exerts immunomodulatory actions. Intermittent administration of PTH and 1,25-dihydroxyvitamin D_3_ has been shown to suppress pro-inflammatory cytokines such as tumor necrosis factor-α (TNF-α) and interleukin-6 (IL-6), particularly in postmenopausal women. Consequently, vitamin D and PTH influence both bone remodeling and inflammatory pathways relevant to periodontal and systemic bone loss [52,53].

Butyrate, a short-chain fatty acid (SCFA), has recently gained attention for its potential role in oral and systemic health. It is produced by commensal gut microbiota through fermentation of dietary fibers and is naturally present in fiber-rich foods, including dairy products, fruits, and vegetables. However, its role in periodontal disease appears context-dependent and remains complex. During active periodontitis, butyrate is locally produced by periodontal pathogens such as *Porphyromonas gingivalis* and *Fusobacterium nucleatum*, and high local concentrations may exert detrimental effects on periodontal tissues. Consequently, topical or excessive exposure to butyrate during active periodontal inflammation may have adverse outcomes [54,55].

In contrast, butyrate confers significant systemic health benefits. SCFA-producing bacteria, including *Lactobacillus* and *Bifidobacterium* species, generate butyrate through fermentation of dietary polysaccharides and proteins. After absorption in the colon, butyrate enhances gut barrier integrity, promotes anti-inflammatory immune responses, and reduces pathogen-induced hyperinflammation. Oral butyrate supplementation is generally considered safe and well tolerated, with minor gastrointestinal side effects reported at higher doses. Daily doses ranging from 150 to 300 mg have not been associated with significant adverse effects [56].

Mechanistically, butyrate influences bone metabolism through multiple pathways. Experimental studies have shown that butyrate induces metabolic reprogramming of osteoclasts, leading to downregulation of tumor necrosis factor receptor–associated factor 6 (TRAF6) and nuclear factor of activated T cells cytoplasmic 1 (NFATc1). Additionally, gut microbiota colonization and SCFA supplementation have been associated with increased insulin-like growth factor-1 (IGF-1) levels, a hormone essential for skeletal development and maintenance of bone mass. Butyrate also promotes the expansion and differentiation of regulatory T cells (Tregs), which suppress osteoclastogenesis and bone resorption. Increased Treg populations in bone marrow and intestinal tissues further stimulate CD8^+^ T cells to produce Wingless-type 10b (WNT10b), a key signaling molecule that enhances bone formation [55,56].

From a broader mechanistic perspective, these findings support the concept of a gut–bone–periodontal axis in which butyrate acts as a long-range regulatory metabolite. Gut-derived butyrate may influence periodontal and alveolar bone homeostasis indirectly through systemic immunomodulation, endocrine signaling (e.g., IGF-1 induction), and regulation of osteoclast–osteoblast balance. Importantly, the effects of butyrate appear to be context-dependent: while physiologic systemic levels may exert anti-inflammatory and bone-protective effects, excessive local concentrations produced during active periodontal infection may contribute to tissue damage. This dialectical role underscores the complexity of butyrate signaling and highlights the need for future mechanistic and translational studies to delineate dose-, site-, and disease-specific effects within the gut–bone–periodontal axis.

### 4.4. Smoking, Periodontal Diseases and BMD

Smoking represents another important shared risk factor for both periodontitis and osteoporosis and contributes to bone loss through multiple biological mechanisms. Cigarette smoking adversely affects periodontal tissues by altering immune responses and bone remodeling pathways. Although findings regarding the influence of smoking on gingival crevicular fluid cytokine profiles remain inconsistent, evidence consistently demonstrates suppression of osteoprotegerin (OPG) levels in both gingival crevicular fluid and serum among smokers. This imbalance results in an increased receptor activator of nuclear factor kappa-B ligand (RANKL)/OPG ratio, which directly promotes osteoclastic activity and accelerates bone resorption in smoking-associated periodontitis.

In addition to its effects on RANKL-mediated bone loss, smoking induces oxidative stress through increased production of reactive oxygen species (ROS) within gingival tissues. Elevated oxidative stress further amplifies inflammatory responses and contributes to degradation of the periodontal supporting structures, thereby exacerbating alveolar bone loss [57,58,59,60,61].

## 5. Therapeutic Considerations and Treatment Modalities

Therapeutic considerations and treatment modalities for periodontal disease in osteoporotic patients are increasingly informed by advances in the understanding of the underlying pathological mechanisms discussed earlier in this review, including chronic inflammation, immune dysregulation, altered bone remodeling, and host–microbiome interactions. Contemporary strategies aim not only to control periodontal inflammation but also to modulate osteoclast–osteoblast balance, enhance alveolar bone regeneration, and mitigate the impact of systemic bone loss on oral tissues.

Several pharmacological agents commonly used in the management of osteoporosis, including bisphosphonates, raloxifene, denosumab, teriparatide (TPTD), and romosozumab, have been shown to improve jaw bone mineral density (BMD). By modulating bone remodeling pathways, these agents may also contribute to improvements in periodontal stability. Most anti-osteoporotic medications exert their effects through anti-resorptive mechanisms; however, medication-related osteonecrosis of the jaw (MRONJ), particularly associated with bisphosphonates, remains a clinically significant adverse outcome that necessitates careful dental risk assessment and monitoring [62,63].

Denosumab and raloxifene reduce jaw bone resorption through inhibition of the receptor activator of nuclear factor kappa-B ligand (RANKL) pathway and estrogen receptor modulation, respectively. While these mechanisms directly target osteoclast-mediated bone loss, both agents carry a potential risk for jaw osteonecrosis. Furthermore, discontinuation of denosumab has been associated with a transient rebound increase in bone turnover and accelerated bone loss, underscoring the importance of coordinated medical–dental management in treated patients [64,65].

Romosozumab, a humanized monoclonal antibody targeting sclerostin, represents an anabolic therapeutic approach that enhances bone formation by removing sclerostin-mediated inhibition of osteogenesis. Experimental studies have demonstrated its capacity to promote periodontal bone regeneration following periodontitis and to improve peri-implant osseointegration, highlighting its potential relevance to alveolar bone repair. Nevertheless, its clinical use requires caution due to reported cardiovascular risks, which may limit its broader application [66,67,68,69,70].

Beyond conventional pharmacotherapy, biologically active compounds derived from dietary and herbal sources have gained interest for their potential to modulate bone metabolism and periodontal inflammation. Phytoestrogens are naturally occurring nonsteroidal polyphenolic compounds with structural and functional similarities to estradiol. Isoflavonoids, the most prevalent class, are metabolized in the intestine into active forms such as genistein and daidzein [71]. Experimental studies indicate that puerarin, an isoflavone compound, exerts protective effects against mandibular osteoporosis by preserving bone morphometric parameters and BMD, particularly when combined with zinc. Other flavonoids, including myricetin, polymethoxyflavonoids, and rutin, have demonstrated dose-dependent suppression of alveolar bone loss and osteoclast activity without significant adverse effects, suggesting therapeutic potential in jaw osteoporosis and periodontal bone preservation [72,73].

Herbal-derived compounds have also been explored for their anti-osteoporotic and anti-inflammatory properties. Curcumin, the active component of *Curcuma longa*, has demonstrated beneficial effects in experimental osteoporosis models. CMC2.24, a chemically modified curcumin, inhibits pro-inflammatory mediators—particularly matrix metalloproteinases (MMPs)—and reduces bone resorption, especially in diabetic osteoporosis. Similarly, tanshinone, derived from *Salvia miltiorrhiza* (Danshen), has been shown to restore alveolar bone microarchitecture and improve bone marrow stromal cell (BMSC) aging in animal models, linking host-modulatory effects with bone regenerative potential [74,75].

Emerging evidence also highlights the role of gut microbiota modulation in skeletal and periodontal health. Berberine, an alkaloid extracted from medicinal plants, has demonstrated bone-protective effects through modulation of gut microbiota composition and systemic inflammatory signaling. In ovariectomized rat models with periodontitis, berberine administration reduced serum lipopolysaccharide (LPS) levels and inflammatory mediators, resulting in improved alveolar bone levels and periodontal attachment gain, thereby illustrating the relevance of gut–bone–periodontal interactions in therapeutic design [76].

Regenerative approaches based on extracellular vesicles (EVs) have gained attention for their ability to target key pathological pathways involved in bone loss and tissue repair. EVs are membrane-bound particles released by various cell types and are classified into apoptotic bodies, microvesicles, and exosomes. Among these, exosomes have been extensively studied for their role in maintaining oral bone homeostasis [77,78]. Stem cell–derived EVs have demonstrated regenerative potential by supporting osteoblast differentiation and suppressing osteoclast activity. For example, BMSC-derived exosomes containing metastasis-associated lung adenocarcinoma transcript 1 (MALAT1) enhance osteoblast activity, while long non-coding RNA taurine upregulated gene 1 (lncTUG1) has been associated with improved fracture healing. Experimental studies further indicate that BMSC-derived EVs can restore alveolar bone structure and calcium levels in glucocorticoid-induced osteoporotic models [79,80].

Milk-derived extracellular vesicles (MEVs) have also shown beneficial effects in ovariectomized animal models by reducing osteoclast numbers and increasing osteoblast and osteocyte activity. These effects are partly mediated through modulation of the RANKL/OPG ratio and endogenous microRNAs, such as miR-34a, which suppress RANKL expression. Despite encouraging findings, challenges related to targeted delivery, dosage optimization, and long-term stability must be addressed before EV-based therapies can be translated into clinical practice for jaw osteoporosis [81,82,83,84].

In addition to pharmacological and molecular approaches, mechanical and physical stimulation modalities have emerged as adjunctive strategies for managing osteoporosis. Bone adapts to dynamic mechanical loading by altering its mass, microstructure, and strength, and weight-bearing exercise has been shown to improve bone density in osteoporotic individuals [85,86]. In the craniofacial region, where physiological loading is limited, localized mechanical stimulation techniques have been investigated. High-frequency acceleration (HFA) applied to alveolar bone has demonstrated anabolic effects by enhancing bone formation and reducing catabolic activity [87,88].

Low-level laser therapy (LLLT) represents another local intervention that influences cellular behavior through photophysical, photochemical, and photobiological mechanisms. LLLT has been shown to enhance bone regeneration and calcium deposition in osteoporotic jaw models, including improved bone formation around orthodontic mini-implants and enhanced alveolar bone remodeling in diabetic and ovariectomized rats. Therapeutic outcomes appear to be energy-density dependent, with optimal effects observed at approximately 640 J/cm^2^ [89,90,91,92,93].

Pulsed electromagnetic field (PEMF) therapy is an emerging physical modality that stimulates osteoblastic activity and improves bone quality and quantity. In addition to its effects on bone metabolism, PEMF has been shown to reduce periodontal pathogenic bacterial load and inflammatory cytokine levels, suggesting potential benefits for patients with concomitant osteoporosis and periodontitis [94].

Collectively, these therapeutic strategies illustrate how mechanistic insights into inflammation, immune regulation, bone metabolism, and host–microbiome interactions are increasingly being translated into targeted interventions for managing periodontal disease and alveolar bone loss in osteoporotic patients. While these approaches show promise as adjunctive therapies, further translational and clinical studies are required to establish their long-term safety, efficacy, and clinical applicability.

## 6. Clinical Implications

Jaw osteoporosis has a significant impact on oral health and influences the prognosis and outcomes of various dental procedures, including periodontal therapy, orthodontic treatment, dental implant placement, and tooth extraction. Given the established association between systemic osteoporosis and oral bone quality, the placement of dental implants in osteoporotic patients remains a subject of clinical debate. Retrospective studies evaluating dental implant outcomes have demonstrated a relationship between surgeon-assessed jaw bone quality and implant failure rates. Implants placed in high-quality bone exhibited lower failure rates compared with those placed in medium- or low-quality bone. Similarly, meta-analyses have reported higher four-year cumulative success rates for machined-surface and Osseotite implants when inserted into dense, normal bone compared with non-dense bone, underscoring bone quality as a critical determinant of implant success [95,96,97,98,99,100].

Orthodontic mini-implants (OMIs) are also affected by jaw bone density, as their primary stability and anchorage efficiency depend largely on bone quality. Experimental and cadaveric studies have shown that initial OMI stability in the palate is primarily influenced by local bone density and the depth of implant insertion. Because bone mineral density varies across different regions of the jaw, selecting optimal insertion sites and adjusting implantation angles to increase cortical bone contact may enhance OMI stability and success rates [101,102,103].

Delayed wound healing represents another clinically relevant consequence of jaw osteoporosis. Animal studies have demonstrated that age-related jaw osteoporosis adversely affects alveolar socket healing following tooth extraction and delays bone regeneration, with more pronounced effects observed in female rats. In ovariectomized models, accelerated alveolar bone resorption and reduced new bone formation within extraction sockets have been consistently reported [104,105,106,107].

Clinical evidence further supports these findings. Two large-scale studies conducted in Japan evaluated post-extraction wound healing in elderly patients with self-reported histories of vertebral compression fractures or fragility fractures. Both studies reported a higher prevalence of delayed wound healing among individuals with a significant history of osteoporosis-related fractures, highlighting the clinical relevance of systemic bone health in oral surgical outcomes [107].

An overview of the key clinical studies evaluating the association between osteoporosis and periodontal disease, along with their main findings and methodological limitations, is summarized in Table 1. The principal dental and therapeutic implications relevant to periodontal care, implant therapy, and oral surgery in osteoporotic patients are summarized in Table 2.

## 7. Research Gaps and Critical Appraisal

### 7.1. Interpretive Synthesis of the Evidence

Across the reviewed literature, a generally consistent association is observed between reduced systemic bone mineral density and increased radiographic alveolar bone loss, particularly in postmenopausal and elderly populations. However, associations with clinical periodontal parameters such as probing depth and clinical attachment loss are more variable. This discrepancy may reflect differences in outcome sensitivity, as radiographic measures more directly capture cumulative bone remodeling changes influenced by systemic osteoporosis.

Variability in findings across studies is likely attributable to heterogeneity in osteoporosis definitions, periodontal case criteria, and adjustment for shared confounders, including smoking, diabetes, age, menopausal status, and medication exposure. Differences in jaw site assessment, implant location, and follow-up duration further complicate direct comparison. Importantly, while mechanistic evidence supports a biologically plausible bidirectional interaction between bone metabolism and periodontal inflammation, the predominance of cross-sectional designs limits causal inference. These findings highlight the need for longitudinal and interventional studies using standardized definitions and clinically relevant endpoints to better inform dental management strategies in osteoporotic patients. Together, these findings suggest that the periodontal relevance of osteoporosis may be outcome-dependent, with radiographic bone measures capturing systemic skeletal effects more reliably than traditional clinical periodontal parameters.

### 7.2. Critical Appraisal of the Current Evidence

As summarized in Table 1 and Table 2, the existing body of evidence is largely observational and heterogeneous, with substantial variability in study design, diagnostic definitions, outcome measures, and confounder adjustment.

Although the literature increasingly supports an association between systemic bone loss and periodontal breakdown, much of the available evidence remains observational and largely cross-sectional, limiting causal inference and the ability to confirm temporality. Substantial heterogeneity exists across studies in osteoporosis definitions (DXA site, thresholds, fracture history), periodontitis case definitions, and radiographic approaches for alveolar bone assessment, which contributes to inconsistent clinical estimates.

Residual confounding remains a major concern, as key shared determinants—including age, smoking exposure, diabetes, body mass index, vitamin D status, menopausal stage, and medication history—are inconsistently measured or incompletely adjusted. Notably, associations appear more consistent for radiographic alveolar bone outcomes than for clinical parameters such as probing depth or attachment levels, suggesting differential sensitivity of outcome measures across studies.

Several factors may underlie these discrepancies. Radiographic measures directly capture mineralized alveolar bone changes and may therefore be more sensitive to systemic alterations in bone metabolism associated with osteoporosis. In contrast, clinical periodontal parameters reflect soft-tissue inflammation and are influenced by examiner variability, local plaque control, and short-term inflammatory fluctuations. Moreover, osteoporosis-related bone remodeling changes may precede or occur independently of overt clinical periodontal deterioration, particularly in cross-sectional study designs. Collectively, these methodological and biological differences represent a key unresolved controversy, raising the possibility that systemic osteoporosis preferentially affects alveolar bone architecture detectable radiographically, while conventional clinical periodontal measures may be less sensitive to systemic skeletal changes.

Evidence regarding the impact of osteoporosis therapies on periodontal and implant outcomes remains evolving. Anti-resorptive agents may improve systemic and jaw bone density but introduce clinically relevant concerns (e.g., MRONJ risk, rebound bone loss after denosumab cessation).

Importantly, the strength of available evidence varies substantially across study types. Much of the mechanistic insight into the osteoporosis–periodontitis relationship is derived from animal and in vitro models, which provide valuable biological plausibility but limited direct clinical generalizability. Human studies are predominantly cross-sectional or observational, restricting causal inference and susceptibility to residual confounding. Interventional clinical trials specifically designed to evaluate periodontal outcomes in osteoporotic populations remain scarce. Accordingly, clinical implications drawn from the current literature should be interpreted cautiously and viewed as hypothesis-generating rather than definitive.

Overall, well-designed longitudinal cohorts and controlled clinical studies with standardized definitions and comprehensive confounder adjustment are needed to clarify causality, quantify effect sizes, and guide dental management in osteoporotic patients.

### 7.3. Recommendations and Future Directions

Despite growing evidence, a gap remains in fully elucidating the relationship between periodontitis and osteoporosis, particularly regarding the clinical significance of radiographic versus clinical periodontal outcomes. In addition, the dental implications of various osteoporosis treatment modalities—especially their effects on dental implant placement, osseointegration, and long-term implant survival—remain incompletely understood and warrant further investigation.

While experimental and observational evidence supports a biologically plausible association between osteoporosis and periodontal disease, definitive clinical recommendations require validation through well-designed longitudinal and interventional studies. Future research should prioritize standardized outcome measures, rigorous confounder control, and prospective study designs to inform evidence-based dental management strategies in osteoporotic patients.

## 8. Conclusions

Current evidence supports a meaningful and potentially bidirectional association between osteoporosis and periodontal disease, driven by shared inflammatory pathways, common risk factors, and altered bone remodeling. Reduced systemic bone mineral density appears to be more consistently associated with radiographic alveolar bone loss than with clinical periodontal parameters, highlighting the importance of outcome selection in future studies. Conversely, chronic periodontal inflammation may contribute to systemic inflammatory burden with potential relevance to skeletal health.

From a clinical perspective, jaw osteoporosis has relevant implications for periodontal therapy, dental implant placement, orthodontic anchorage, and post-extraction healing. Dentists are therefore well positioned to contribute to early identification of patients at risk for low bone density through careful oral assessment, risk factor modification, and timely referral for fracture risk evaluation.

Despite growing mechanistic and clinical evidence, the literature remains limited by heterogeneity in study design, diagnostic criteria, and confounder control. Well-designed longitudinal and interventional studies with standardized definitions are needed to clarify causality, quantify clinical risk, and guide evidence-based dental management strategies for patients with osteoporosis.

## Figures and Tables

**Table 1:** Key Clinical Studies Evaluating the Association between Osteoporosis and Periodontal Disease.

Author (Year)	Study Design	Population	Osteoporosis Assessment	Periodontal Outcomes	Main Findings	Key Limitations
Jeffcoat et al. [108].	Cross-sectional	Postmenopausal women	DXA (hip/spine)	Radiographic ABL	Lower BMD associated with increased alveolar bone loss	Cross-sectional design
Ronderos et al. [109].	Cross-sectional	Postmenopausal women	DXA	CAL, PD, ABL	Osteoporosis linked to worse periodontal parameters	Confounding by age
Inagaki et al. [110].	Cohort	Elderly adults	Self-reported fractures	Tooth loss, healing	Delayed wound healing in osteoporotic patients	Self-reported data
Tezal et al. [111].	Cross-sectional	Postmenopausal women	BMD proxy indices	Radiographic ABL	Significant association with low BMD	Indirect BMD measures
Meta-analyses [43,44,45]	Systematic review/meta-analysis	Postmenopausal vs. controls	DXA	CAL, PD, ABL	Worse periodontal parameters in osteoporotic group	Study heterogeneity

**Table 2:** Dental and Therapeutic Implications of Osteoporosis and Its Management.

Topic	Study Type	Key Findings	Clinical Implications
Dental implants	Retrospective studies	Higher failure rates in low-quality bone	Bone quality should guide implant planning
Implant surface studies	Meta-analysis	Higher success in dense bone	Importance of site selection
Orthodontic mini-implants	Cadaver and animal studies	Stability depends on local BMD and cortical thickness	Site/angle modification recommended
Tooth extraction healing	Animal and clinical studies	Delayed healing in osteoporotic patients	Careful post-extraction monitoring
Anti-resorptive therapy	Clinical reviews	Improved BMD but MRONJ risk	Risk–benefit assessment needed
Denosumab cessation	Observational data	Rebound bone loss	Timing of dental surgery is critical

## Data Availability

No new data were created or analyzed in this study. Data sharing is not applicable to this article.

## References

[1] Balto K.A., Gomaa M.M., Feteih R.M., AlAmoudi N.M., Elsamanoudy A.Z., Hassanien M.A., Ardawi M.-S.M. (2018). Dental panoramic radiographic indices as a predictor of osteoporosis in postmenopausal Saudi women. J. Bone Metab..

[2] Qaseem A., Forciea M.A., McLean R.M., Denberg T.D. (2017). Treatment of Low Bone Density or Osteoporosis to Prevent Fractures in Men and Women: A Clinical Practice Guideline Update From the American College of Physicians. Ann. Intern. Med..

[3] Mishra G.D., Davies M.C., Hillman S., Chung H.F., Roy S., Maclaran K., Hickey M. (2024). Optimising health after early menopause. Lancet.

[4] Di Stefano M., Polizzi A., Santonocito S., Romano A., Lombardi T., Isola G. (2022). Impact of oral microbiome in periodontal health and periodontitis: A critical review on prevention and treatment. Int. J. Mol. Sci..

[5] Sczepanik F.S.C., Grossi M.L., Casati M., Goldberg M., Glogauer M., Fine N., Tenenbaum H.C. (2020). Periodontitis is an inflammatory disease of oxidative stress: We should treat it that way. Periodontol. 2000.

[6] Wang C.J., McCauley L.K. (2016). Osteoporosis and periodontitis. Curr. Osteoporos. Rep..

[7] Vieira A.R., Albandar J.M. (2014). Role of genetic factors in the pathogenesis of aggressive periodontitis. Periodontol. 2000.

[8] Albandar J.M., Susin C., Hughes F.J. (2018). Manifestations of systemic diseases and conditions that affect the periodontal attachment apparatus: Case definitions and diagnostic considerations. J. Periodontol..

[9] Caton J.G., Armitage G., Berglundh T., Chapple I.L., Jepsen S., Kornman K.S., Mealey B.L., Papapanou P.N., Sanz M., Tonetti M.S. (2018). A new classification scheme for periodontal and peri-implant diseases and conditions—Introduction and key changes from the 1999 classification. J. Periodontol..

[10] Heuchert J., Kozieł S., Spinek A.E. (2024). Radiomorphometric indices of the mandible as indicators of decreased bone mineral density and osteoporosis—Meta-analysis and systematic review. Osteoporos. Int..

[11] Brennan R.M., Genco R.J., Hovey K.M., Trevisan M., Wactawski-Wende J. (2007). Clinical attachment loss, systemic bone density, and subgingival calculus in postmenopausal women. J. Periodontol..

[12] Ishii K., Taguchi A., Nakamoto T., Ohtsuka M., Sutthiprapaporn P., Tsuda M., Kodama I., Kudo Y., Sumida H., Suei Y. (2007). Diagnostic efficacy of alveolar bone loss of the mandible for identifying postmenopausal women with femoral osteoporosis. Dentomaxillofac. Radiol..

[13] Taguchi A., Ohtsuka M., Tsuda M., Nakamoto T., Kodama I., Inagaki K., Noguchi T., Kudo Y., Suei Y., Tanimoto K. (2007). Risk of vertebral osteoporosis in post-menopausal women with alterations of the mandible. Dentomaxillofac. Radiol..

[14] Takaishi Y., Okamoto Y., Ikeo T., Morii H., Takeda M., Hide K., Arai T., Nonaka K. (2005). Correlations between periodontitis and loss of mandibular bone in relation to systemic bone changes in postmenopausal Japanese women. Osteoporos. Int..

[15] Ayed M.S., Shafiq S.S., Diab H.M., Alahmari A.D., Divakar D.D. (2018). Assessing periapical dental radiographs as a screening parameter for early indications of osteoporosis in postmenopausal periodontal patients and root surface evaluation using spectrochemical analysis. Saudi Med. J..

[16] Jonasson G., Bankvall G., Kiliaridis S. (2001). Estimation of skeletal bone mineral density by means of the trabecular pattern of the alveolar bone, its interdental thickness, and the bone mass of the mandible. Oral Surg. Oral Med. Oral Pathol. Oral Radiol. Endodontol..

[17] Penoni D.C., Vettore M.V., Torres S.R., Farias M.L.F., Leão A.T.T. (2019). An investigation of the bidirectional link between osteoporosis and periodontitis. Arch. Osteoporos..

[18] Park J.-H., Park M.-S., Kim H.-J., Lee H., Kim J.-W., Song T.-J. (2023). Better oral hygiene is associated with a reduced risk of osteoporotic fracture: A nationwide cohort study. Front. Endocrinol..

[19] Jonasson G., Sundh V., Hakeberg M., Hassani-Nejad A., Lissner L., Ahlqwist M. (2013). Mandibular bone changes in 24 years and skeletal fracture prediction. Clin. Oral Investig..

[20] Jonasson G., Sundh V., Ahlqwist M., Hakeberg M., Björkelund C., Lissner L. (2011). A prospective study of mandibular trabecular bone to predict fracture incidence in women: A low-cost screening tool in the dental clinic. Bone.

[21] Hassani-Nejad A., Ahlqwist M., Hakeberg M., Jonasson G. (2013). Mandibular trabecular bone as fracture indicator in 80-year-old men and women. Eur. J. Oral Sci..

[22] Lindh C., Horner K., Jonasson G., Olsson P., Rohlin M., Jacobs R., Karayianni K., van der Stelt P., Adams J., Marjanovic E. (2008). The use of visual assessment of dental radiographs for identifying women at risk of having osteoporosis: The OSTEODENT project. Oral Surg. Oral Med. Oral Pathol. Oral Radiol. Endodontol..

[23] Pacios S., Xiao W., Mattos M., Lim J., Tarapore R.S., Alsadun S., Yu B., Wang C.-Y., Graves D.T. (2015). Osteoblast lineage cells play an essential role in periodontal bone loss through activation of nuclearfactor-kappaB. Sci. Rep..

[24] Chang J., Wang Z., Tang E., Fan Z., McCauley L., Franceschi R., Guan K., Krebsbach P.H., Wang C.-Y. (2009). Inhibition of osteoblastic bone formation by nuclear factor-kappaB. Nat. Med..

[25] Haugeberg G., Conaghan P.G., Quinn M., Emery P. (2009). Bone loss in patients with active early rheumatoid arthritis: Infliximab and methotrexate compared with methotrexate treatment alone. Explorative analysis from a 12-month randomised, double-blind, placebo-controlled study. Ann. Rheum. Dis..

[26] Sirisereephap K., Maekawa T., Tamura H., Hiyoshi T., Domon H., Isono T., Terao Y., Maeda T., Tabeta K. (2022). Osteoimmunology in periodontitis: Local proteins and compounds to alleviate periodontitis. Int. J. Mol. Sci..

[27] Hajishengallis G., Reis E.S., Mastellos D.C., Ricklin D., Lambris J.D. (2017). Novel mechanisms and functions of complement. Nat. Immunol..

[28] Zhang X., Kimura Y., Fang C., Zhou L., Sfyroera G., Lambris J.D., Wetsel R.A., Miwa T., Song W.-C. (2007). Regulation of Toll-like receptor–mediated inflammatory response by complement in vivo. Blood.

[29] Hajishengallis G., Kajikawa T., Hajishengallis E., Maekawa T., Reis E.S., Mastellos D.C., Yancopoulou D., Hasturk H., Lambris J.D. (2019). Complement-dependent mechanisms and interventions in periodontal disease. Front. Immunol..

[30] Patters M., Niekrash C., Lang N.P. (1989). Assessment of complement cleavage in gingival fluid during experimental gingivitis in man. J. Clin. Periodontol..

[31] Li Y., Ling J., Jiang Q. (2021). Inflammasomes in alveolar bone loss. Front. Immunol..

[32] López-Otín C., Blasco M.A., Partridge L., Serrano M., Kroemer G. (2013). The hallmarks of aging. Cell.

[33] Mundy G.R. (2007). Osteoporosis and inflammation. Nutr. Rev..

[34] Persson G.R. (2018). Periodontal complications with age. Periodontol. 2000.

[35] Ebersole J.L., Steffen M.J., Gonzalez-Martinez J., Novak M.J. (2008). Effects of age and oral disease on systemic inflammatory and immune parameters in nonhuman primates. Clin. Vaccine Immunol..

[36] Gil-Montoya J.A., Garrido-Martínez M., Barrios-Rodríguez R., Ramos-García P., Lenouvel D., Montes-Castillo C., Martínez-Ramírez M.J. (2021). Association between low bone mineral density and periodontitis in generally healthy perimenopausal women. J. Periodontol..

[37] Penoni D.C., Torres S.R., Farias M.L., Fernandes T.M., Luiz R.R., Leão A.T. (2016). Association of osteoporosis and bone medication with the periodontal condition in elderly women. Osteoporos. Int..

[38] Penoni D.C., Fidalgo T.K., Torres S.R., Varela V.M., Masterson D., Leão A.T., Maia L.C. (2017). Bone Density and Clinical Periodontal Attachment in Postmenopausal Women: A Systematic Review and Meta-Analysis. J. Dent. Res..

[39] Mohebbi R., Shojaa M., Kohl M., von Stengel S., Jakob F., Kerschan-Schindl K., Lange U., Peters S., Thomasius F., Uder M. (2023). Exercise training and bone mineral density in postmenopausal women: An updated systematic review and meta-analysis of intervention studies with emphasis on potential moderators. Osteoporos. Int..

[40] Yu B., Wang C.-Y. (2022). Osteoporosis and periodontal diseases—An update on their association and mechanistic links. Periodontol. 2000.

[41] Taguchi A., Urano T., Nakamura Y., Shiraki M. (2023). Increased risk of tooth loss in postmenopausal women with fractures/low bone status. JBMR Plus.

[42] Qi J., Chen J., Pang Y., Guo Y., Chen G., Liu Y., Wang J., Liu E. (2023). Association between periodontal disease and osteoporosis in postmenopausal women: A systematic review and meta-analysis. Heliyon.

[43] Tilotta F., Gosset M., Herrou J., Briot K., Roux C. (2025). Association between osteoporosis and periodontitis. Jt. Bone Spine.

[44] Alayash Z., Baumeister S.-E., Reckelkamm S.L., Holtfreter B., Kocher T., Baurecht H., Ehmke B., Nolde M. (2023). Association between total body bone mineral density and periodontal disease (meta-analysis/evidence synthesis). J. Periodontol..

[45] Berg G., Ekerfelt C., Hammar M., Lindgren R., Matthiesen L., Ernerudh J. (2002). Cytokine changes in postmenopausal women treated with estrogens: A placebo-controlled study. Am. J. Reprod. Immunol..

[46] Botelho J., Machado V., Proença L., Delgado A.S., Mendes J.J. (2020). Vitamin D Deficiency and Oral Health: A Comprehensive Review. Nutrients.

[47] Bischoff-Ferrari H.A., Willett W.C., Wong J.B., Giovannucci E., Dietrich T., Dawson-Hughes B. (2005). Fracture prevention with vitamin D supplementation: A meta-analysis of randomized controlled trials. JAMA.

[48] Liu X., Dai B., Chuai Y., Hu M., Zhang H. (2023). Associations between vitamin D levels and periodontal attachment loss. Clin. Oral Investig..

[49] Al-Zahrani M.S. (2006). Increased intake of dairy products is related to lower periodontitis prevalence. J. Periodontol..

[50] Zhou F., Ma N., Su R., He X., Wang X., Zhou Y., Shi J. (2021). Serum 25-hydroxyvitamin D is negatively associated with severe periodontitis: A cross-sectional study. BMC Oral Health.

[51] Wojda S.J., Donahue S.W. (2018). Parathyroid hormone for bone regeneration. J. Orthop. Res..

[52] Li Q., Lyu C.H., Chen L., Liu X., Wang H., Sun W. (2020). Synthetic PTHrP1-36 and its role in promoting alveolar bone formation. Shanghai J. Stomatol..

[53] Quintero S., Ait-Aissa K., Munkhsaikhan U., Sahyoun A.M., Apu E.H., Abidi A.H., Kassan M. (2025). Exploring the relationship between periodontal diseases and osteoporosis: Potential role of butyrate. Biomed. Pharmacother..

[54] Banasiewicz T., Domagalska D., Borycka-Kiciak K., Rydzewska G. (2020). Determination of butyric acid dosage based on clinical and experimental studies—A literature review. Gastroenterol. Rev..

[55] Tyagi A.M., Yu M., Darby T.M., Vaccaro C., Li J.Y., Owens J.A., Hsu E., Adams J., Weitzmann M.N., Jones R.M. (2018). The microbial metabolite butyrate stimulates bone formation via t regulatory cell-mediated regulation of WNT10B expression. Immunity.

[56] Buduneli N., Buduneli E., Kütükçüler N. (2009). Interleukin-17, RANKL, and osteoprotegerin levels in gingival crevicular fluid from smoking and non-smoking patients with chronic periodontitis during initial periodontal treatment. J. Periodontol..

[57] César-Neto J.B., Duarte P.M., de Oliveira M.C., Tambeli C.H., Sallum E.A., Nociti F.H. (2007). Smoking modulates interleukin-6: Interleukin-10 and RANKL:osteoprotegerin ratios in the periodontal tissues. J. Periodontal Res..

[58] Lappin D.F., Sherrabeh S., Jenkins W.M., Macpherson L.M. (2007). Effect of smoking on serum RANKL and OPG in sex, age and clinically matched supportive-therapy periodontitis patients. J. Clin. Periodontol..

[59] Tang T.H., Fitzsimmons T.R., Bartold P.M. (2009). Effect of smoking on concentrations of receptor activator of nuclear factor kappa B ligand and osteoprotegerin in human gingival crevicular fluid. J. Clin. Periodontol..

[60] Aziz A.S., Kalekar M.G., Suryakar A.N., Benjamin T., Prakashan M.J., Ahmed B.M.N., Sayyad M. (2013). Assessment of some biochemical oxidative stress markers in male smokers with chronic periodontitis. Indian J. Clin. Biochem..

[61] Lucas S., Omata Y., Hofmann J., Böttcher M., Iljazovic A., Sarter K., Albrecht O., Schulz O., Krishnacoumar B., Krönke G. (2018). Short-chain fatty acids regulate systemic bone mass and protect from pathological bone loss. Nat. Commun..

[62] Yan J., Herzog J.W., Tsang K., Brennan C.A., Bower M.A., Garrett W.S., Sartor B.R., Aliprantis A.O., Charles J.F. (2016). Gut microbiota induce IGF-1 and promote bone formation and growth. Proc. Natl. Acad. Sci. USA.

[63] Ferrari S., Langdahl B. (2023). Mechanisms underlying the long-term and withdrawal effects of denosumab therapy on bone. Nat. Rev. Rheumatol..

[64] Koth V.S., Salum F.G., de Figueiredo M.A.Z., Cherubini K. (2021). Morphological and immunohistochemical features of tooth extraction sites in rats treated with alendronate, raloxifene, or strontium ranelate. Clin. Oral Investig..

[65] Carpenter K.A., Alkhatib D.O., Dulion B.A., Guirado E., Patel S., Chen Y., George A., Ross R.D. (2023). Sclerostin antibody improves alveolar bone quality in the Hyp mouse model of X-linked hypophosphatemia (XLH). Int. J. Oral Sci..

[66] Liu M., Kurimoto P., Zhang J., Niu Q.T., Stolina M., Dechow P.C., Feng J.Q., Hesterman J., Silva M.D., Ominsky M.S. (2018). Sclerostin and DKK1 inhibition preserves and augments alveolar bone volume and architecture in rats with alveolar bone loss. J. Dent. Res..

[67] Fixen C., Tunoa J. (2021). Romosozumab: A review of efficacy, safety, and cardiovascular risk. Curr. Osteoporos. Rep..

[68] Wu D., Sun X., Zhao Y., Liu Y., Gan Z., Zhang Z., Chen X., Cao Y. (2023). Strontium Ranelate inhibits osteoclastogenesis through nf-κb-pathway-dependent autophagy. Bioengineering.

[69] Enezei H.H., Al Qabbani A., Ahmad A., Khamis M.F., Hassani A., Hamad H.A. (2020). The Effect of Strontium on Osteoblastogenesis and Osteoclastogenesis in Dental Stem Cells-induced Epidermal Growth Factor at Molecular Level: In Vitro Study. J. Hard Tissue Biol..

[70] Enezei H.H. (2018). Anti-Resorptive Agent Coupled with IGF-1 Supplementation Modulates Cx43 mRNA Expression in a Maxillofacial Bone Cell Line Culture. Int. Med. J..

[71] Marins L.M., Napimoga M.H., Malta F.S., Miranda T.S., Nani E.P., Franco B., da Silva H.D.P., Duarte P.M. (2020). Effects of strontium ranelate on ligature-induced periodontitis in estrogen-deficient and estrogen-sufficient rats. J. Periodontal Res..

[72] Liu H., Li W., Jia S., Li B. (2017). Puerarin and zinc additively prevent mandibular bone loss through inhibiting osteoclastogenesis in ovariectomized rats. Histol. Histopathol..

[73] Matsumoto S., Tominari T., Matsumoto C., Yoshinouchi S., Ichimaru R., Watanabe K., Hirata M., Grundler F.M.W., Miyaura C., Inada M. (2018). Effects of polymethoxyflavonoids on bone loss induced by estrogen deficiency and by LPS-dependent inflammation in mice. Pharmacruticals.

[74] Huang J., Wu C., Tian B., Zhou X., Ma N., Qian Y. (2016). Myricetin prevents alveolar bone loss in an experimental ovariectomized mouse model of periodontitis. Int. J. Mol. Sci..

[75] Bhatt H.D., Golub L.M., Lee H.M., Kim J., Zimmerman T., Deng J., Hong H., Johnson F., Gu Y. (2023). Efficacy of a novel pleiotropic MMP-inhibitor, CMC2.24, in a long-term diabetes rat model with severe hyperglycemia-induced oral bone loss. J. Inflamm. Res..

[76] Deng J., Golub L.M., Lee H.M., Raja V., Johnson F., Kucine A., Lee W., Xu T.M., Gu Y. (2021). A novel modified-curcumin promotes resolvin-like activity and reduces bone loss in diabetes-induced experimental periodontitis. J. Inflamm. Res..

[77] Jia X., Jia L., Mo L., Yuan S., Zheng X., He J., Chen V., Guo Q., Zheng L., Yuan Q. (2019). Berberine ameliorates periodontal bone loss by regulating gut microbiota. J. Dent. Res..

[78] Chen Y., Huang Y., Li J., Jiao T., Yang L. (2024). Enhancing osteoporosis treatment with engineered mesenchymal stem cell-derived extracellular vesicles: Mechanisms and advances. Cell Death Dis..

[79] Liu C., Li Y., Han G. (2022). Advances of mesenchymal stem cells released extracellular vesicles in periodontal bone remodeling. DNA Cell Biol..

[80] Fang F., Yang J., Wang J., Li T., Wang E., Zhang D., Liu X., Zhou C. (2024). The role and applications of extracellular vesicles in osteoporosis. Bone Res..

[81] Sedik A.S., Kawana K.Y., Koura A.S., Mehanna R.A. (2023). Biological effect of bone marrow mesenchymal stem cell-derived extracellular vesicles on the structure of alveolar bone in rats with glucocorticoid-induced osteoporosis. BMC Musculoskelet. Disord..

[82] Huang X., Li Y., Liao H., Luo X., Zhao Y., Huang Y., Zhou Z., Xiang Q. (2023). Research advances on stem cell-derived extracellular vesicles promoting the reconstruction of alveolar bone through RANKL/RANK/OPG pathway. J. Funct. Biomater..

[83] Qiao X., Tang J., Dou L., Yang S., Sun Y., Mao H., Yang D. (2023). Dental pulp stem cell-derived exosomes regulate anti-inflammatory and osteogenesis in periodontal ligament stem cells and promote the repair of experimental periodontitis in rats. Int. J. Nanomed..

[84] Lee A.E., Choi J.G., Shi S.H., He P., Zhang Q.Z., Le A.D. (2023). DPSC-derived extracellular vesicles promote rat jawbone regeneration. J. Dent. Res..

[85] Yi G., Zhang S., Ma Y., Yang X., Huo F., Chen Y., Yang B., Tian W. (2022). Matrix vesicles from dental follicle cells improve alveolar bone regeneration via activation of the PLC/PKC/MAPK pathway. Stem Cell Res. Ther..

[86] Alikhani M., Alikhani M., Alansari S., Almansour A., Hamidaddin M.A., Khoo E., Lopez J.A., Nervina J.M., Nho J.Y., Oliveira S.M. (2019). Therapeutic effect of localized vibration on alveolar bone of osteoporotic rats. PLoS ONE.

[87] Zhang L., Zheng Y.L., Wang R., Wang X.Q., Zhang H. (2022). Exercise for osteoporosis: A literature review of pathology and mechanism. Front. Immunol..

[88] Santinoni C.D., Oliveira H.F., Batista V.E., Lemos C.A., Verri F.R. (2017). Influence of low-level laser therapy on the healing of human bone maxillofacial defects: A systematic review. J. Photochem. Photobiol. B Biol..

[89] Martins B.G., de Moura V.S., Fujii D.N., Garcez A.S., Suzuki S.S. (2022). Photobiomodulation stimulates surrounding bone formation and increases stability of titanium alloy miniscrews in ovariectomized rats. Lasers Med. Sci..

[90] Maia L.G., Alves A.V., Bastos T.S., Moromizato L.S., Lima-Verde I.B., Ribeiro M.A., Gandini Júnior L.G., de Albuquerque-Júnior R.L.C. (2014). Histological analysis of the periodontal ligament and alveolar bone during dental movement in diabetic rats subjected to low-level laser therapy. J. Photochem. Photobiol. B Biol..

[91] Gomes M.F., da Graças Vilela Goulart M., Giannasi L.C., Hiraoka C.M., de Fátima Santana Melo G., de Sousa A.G.V., Nóbrega C.J.P., Zangaro R.A., Salgado M.A.C. (2017). Effects of the GaAlAs diode laser (780 nm) on the periodontal tissues during orthodontic tooth movement in diabetes rats: Histomorphological and immunohistochemical analysis. Lasers Med. Sci..

[92] Gomes M.F., Goulart M., Giannasi L.C., Hiraoka C.M., Melo G.F.S., Zangaro R.A., Nóbrega C.J.P., Salgado M.A.C. (2018). Effects of the photobiomodulation using different energy densities on the periodontal tissues under orthodontic force in rats with type 2 diabetes mellitus. Braz. Oral Res..

[93] Wang A., Ma X., Bian J., Jiao Z., Zhu Q., Wang P., Zhao Y. (2024). Signalling pathways underlying pulsed electromagnetic fields in bone repair. Front. Bioeng. Biotechnol..

[94] Balachandran K., Ramli R., Karsani S.A., Rahman M.A. (2023). Identification of potential biomarkers and small molecule drugs for bisphosphonate-related osteonecrosis of the jaw (BRONJ): An integrated bioinformatics study using big data. Int. J. Mol. Sci..

[95] Yao Y., Kauffmann F., Maekawa S., Sarment L.V., Sugai J.V., Schmiedeler C.A., Doherty E.J., Holdsworth G., Kostenuik P.J., Giannobile W.V. (2020). Sclerostin antibody stimulates periodontal regeneration in large alveolar bone defects. Sci. Rep..

[96] Liu F., Wang X., He Y., Han R., Wang T., Guo Y. (2024). Jaw osteoporosis: Challenges to oral health and emerging perspectives of treatment. Biomed. Pharmacother..

[97] Merheb J., Temmerman A., Rasmusson L., Kubler A., Thor A., Quirynen M. (2016). Influence of skeletal and local bone density on dental implant stability in patients with osteoporosis. Clin. Implant Dent. Relat. Res..

[98] Takekawa T., Moroi A., Gomi K., Takayama A., Yoshizawa K., Ueki K. (2024). Correlation between acquisition of dental implant stability and hounsfield units at dental implant placement. J. Oral Implantol..

[99] Toriya T., Kitahara T., Hyakutake H., Todo M., Takahashi I. (2023). Analysis for predictors of failure of orthodontic mini-implant using patient-specific finite element models. Ann. Biomed. Eng..

[100] Mohlhenrich S.C., Heussen N., Modabber A., Bock A., Holzle F., Wilmes B., Danesh G., Szalma J. (2021). Influence of bone density, screw size and surgical procedure on orthodontic mini-implant placement—Part B: Implant stability. Int. J. Oral Maxillofac. Surg..

[101] Lee D.W., Park J.H., Bay R.C., Choi S.K., Chae J.M. (2021). Cortical bone thickness and bone density effects on miniscrew success rates: A systematic review and metaanalysis. Orthod. Craniofacial Res..

[102] Biguetti C.C., Chandrashekar B.L., Simionato G.B., Momesso N.R., Duarte M.A.H., Rodrigues D.C., Matsumoto M.A. (2023). Influence of age and gender on alveolar bone healing post tooth extraction in 129 Sv mice: A microtomographic, histological, and biochemical characterization. Clin. Oral Investig..

[103] Miranda T.S., Napimoga M.H., De Franco L., Marins L.M., Malta F.S., Pontes L.A., Morelli F.M., Duarte P.M. (2020). Strontium ranelate improves alveolar bone healing in estrogen-deficient rats. J. Periodontol..

[104] Schini M., Vilaca T., Gossiel F., Salam S., Eastell R. (2022). Bone turnover markers: Basic biology to clinical applications. Endocr. Rev..

[105] Niu Q., He J., Wu M., Liu J., Lu X., Zhang L., Jin Z. (2022). Transplantation of bone marrow mesenchymal stem cells and fibrin glue into extraction socket in maxilla promoted bone regeneration in osteoporosis rat. Life Sci..

[106] Taguchi A., Ikegami S., Tokida R., Kamimura M., Sakai N., Horiuchi H., Takahashi J., Kato H. (2020). Fragility fractures and delayed wound healing after toothextraction in Japanese older adults. J. Bone Miner. Metab..

[107] Taguchi A., Kamimura M., Nakamura Y., Sugino N., Ichinose A., Maezumi H., Fukuzawa T., Ashizawa R., Takahara K., Gushiken S. (2016). Delayed wound healing after tooth extraction and selfreported kyphosis in Japanese men and women. Sci. Rep..

[108] Jeffcoat M.K., Lewis C.E., Reddy M.S., Wang C.Y., Redford M. (2000). Post-menopausal bone loss and its relationship to oral bone loss. Periodontol. 2000.

[109] Ronderos M., Jacobs D.R., Himes J.H., Pihlstrom B.L. (2000). Associations of periodontal disease with femoral bone mineral density and estrogen replacement therapy: Cross-sectional evaluation of U.S. adults from NHANES III. J. Clin. Periodontol..

[110] Inagaki K., Kurosu Y., Kamiya T., Kondo F., Yoshinari N., Noguchi T., Krall E.A., Garcia R.I. (2001). Low metacarpal bone density, tooth loss, and periodontal disease in Japanese women. J. Dent. Res..

[111] Tezal M., Wactawski-Wende J., Grossi S.G., Ho A.W., Dunford R., Genco R.J. (2000). The relationship between bone mineral density and periodontitis in postmenopausal women: A cross-sectional study. J. Periodontol..

